# Transvaginal and Transurethral High-Dose-Rate Brachytherapy in a Patient With Malignant Vaginal Melanoma and Urethral Invasion: A Case Report

**DOI:** 10.7759/cureus.84400

**Published:** 2025-05-19

**Authors:** Riki Inagaki, Takaya Inagaki, Ryuki Shimono, Azusa Awaya, Tetsuo Sonomura

**Affiliations:** 1 Department of Radiology, Wakayama Medical University, Wakayama, JPN

**Keywords:** high dose rate brachytherapy, malignant melanoma, transurethral brachytherapy, urethral invasion, vaginal melanoma

## Abstract

A 60-year-old woman presented with abnormal genital bleeding. Colposcopy and cystoscopy revealed black pigmented lesions in the vulva, vaginal vestibule, vaginal fornix, external urethral orifice, and urethral mucosa. The patient was diagnosed with vaginal malignant melanoma and urethral invasion. Because the melanoma was confined to the vagina and urethra, high-dose-rate brachytherapy using Ir-192 was selected. Vaginal lesions were irradiated using a vaginal applicator and the urethral lesions were irradiated by inserting a flexible catheter into the urethral catheter. The total radiation dose was 48 Gy in eight fractions. Pembrolizumab was started one month after radiotherapy. All black pigmented lesions disappeared within six months after brachytherapy. There was no sign of recurrence at 15 months after treatment. Adverse events included Grade 2 perivaginal inflammation at 6 weeks after treatment and Grade 3 vaginal stenosis at 15 months after treatment. The combination of intracavitary irradiation using a vaginal applicator and transurethral irradiation using a flexible catheter within a urethral catheter enabled delivery of an adequate tumor dose.

## Introduction

Mucosal malignant melanoma (MMM) is a rare type of melanoma that arises in mucosal membranes containing melanocytes. It is more common in Asians than in Caucasians [1]. Tomizuka et al. reported that MMM accounts for 14.8% of malignant melanomas in Japanese patients [2]. The 5-year overall survival (OS) rate of patients with cutaneous malignant melanoma was reported to range from 54% to 80%, whereas the 5-year OS rate for MMM ranged from 5% to 25%. Even with complete resection, the recurrence rate remains high, ranging from 50% to 90% [3-5]. Patients with vaginal cancer and urethral invasion often require extensive surgical resection that necessitates urinary diversion [6,7], although some reports have suggested that brachytherapy can help preserve urinary function in patients with urethral cancer [8]. Radiotherapy combined with an immune checkpoint inhibitor (ICI) has also shown promising therapeutic effects [9,10]. The alpha/beta ratio is a radiobiological parameter that reflects the sensitivity of tissues or tumors to fractionated radiation. A low alpha/beta ratio (typically in the range of 1-3 Gy) suggests greater sensitivity to larger radiation doses and hypofractionation. Malignant melanoma has a low alpha/beta ratio of approximately 2.5, and hypofractionated high-dose irradiation is considered to be a suitable approach [11]. Because brachytherapy can deliver a high radiation dose directly to the tumor by inserting the applicator into the cavity or interstitial tissue, it can achieve excellent local control. Moreover, the radiation exposure to the surrounding organs at risk is minimized due to the rapid dose fall-off with increasing distance from the radiation source [12-14]. However, brachytherapy cannot deliver effective radiation doses to areas distant from the applicator. Therefore, an applicator tailored to the extent of tumor invasion is required.

We report the first case of vaginal malignant melanoma with urethral invasion in which local control was achieved using transvaginal and transurethral high-dose-rate intracavitary brachytherapy (HDR-ICBT).

## Case presentation

**Figure 1:**
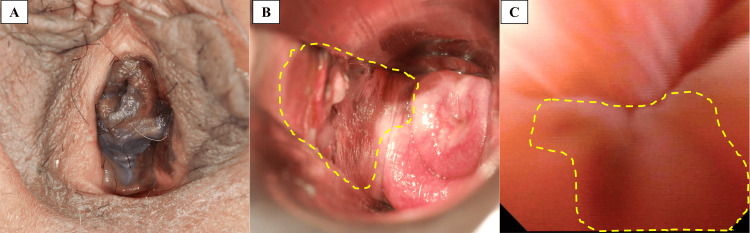
Pre-treatment imaging (A) Macroscopy showing black pigmented lesions extending from the vulva to the external urethral orifice. (B) Colposcopy showing a black pigmented lesion on the vaginal wall (dashed line). (C) Cystoscopy showing a black pigmented lesion on the urethral mucosa between the 4 and 8 o’clock positions (dashed line).

**Figure 2:**
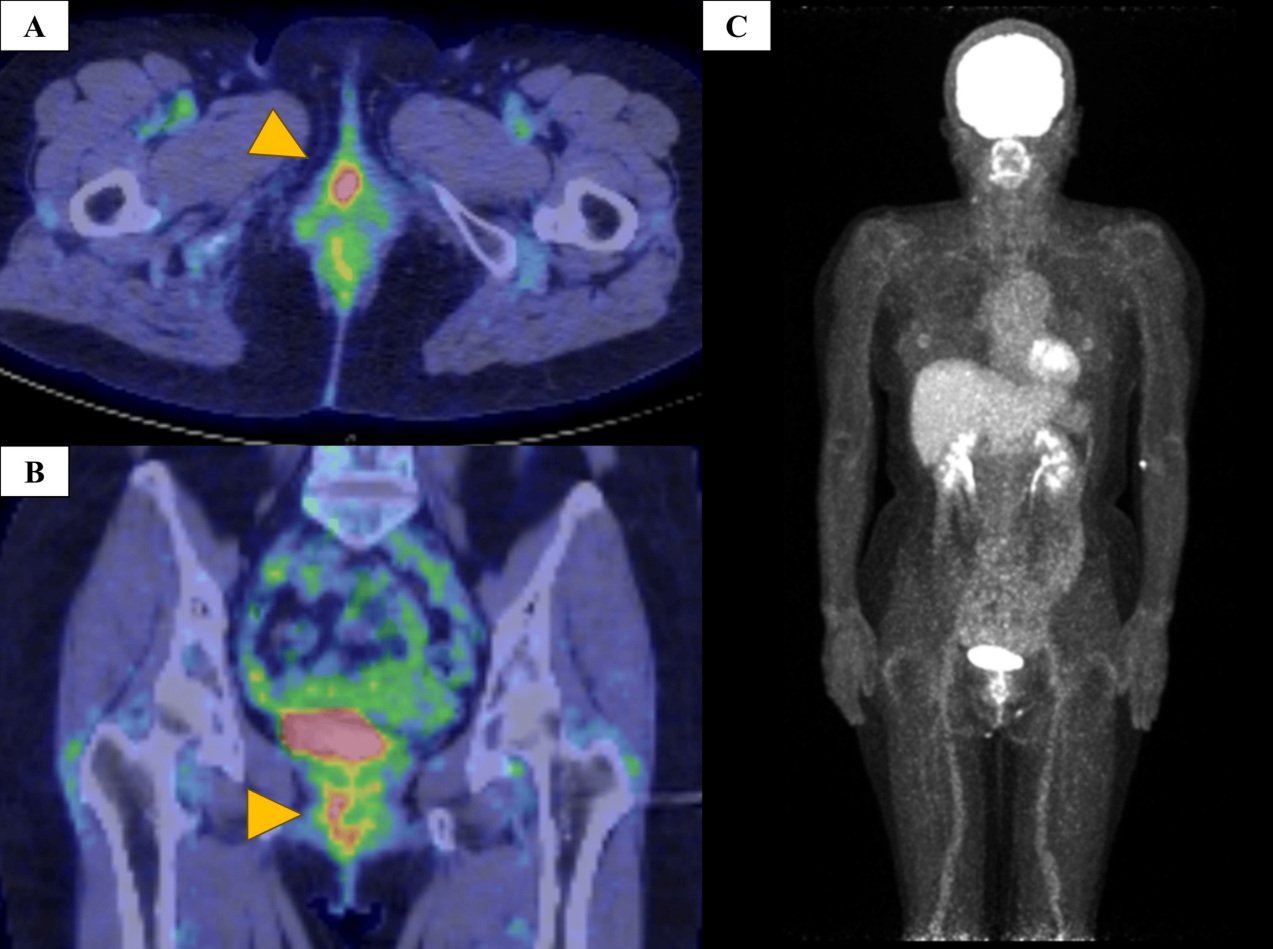
Pre-treatment FDG-PET/CT (A, B) Axial and coronal imaging showing FDG uptake in the vagina and urethra (arrowheads). (C) MIP image showing no lymph node metastasis or distant metastasis. FDG: ^18^fluorodeoxyglucose; PET/CT: positron emission tomography/computed tomography; MIP: maximum intensity projection

**Figure 3:**
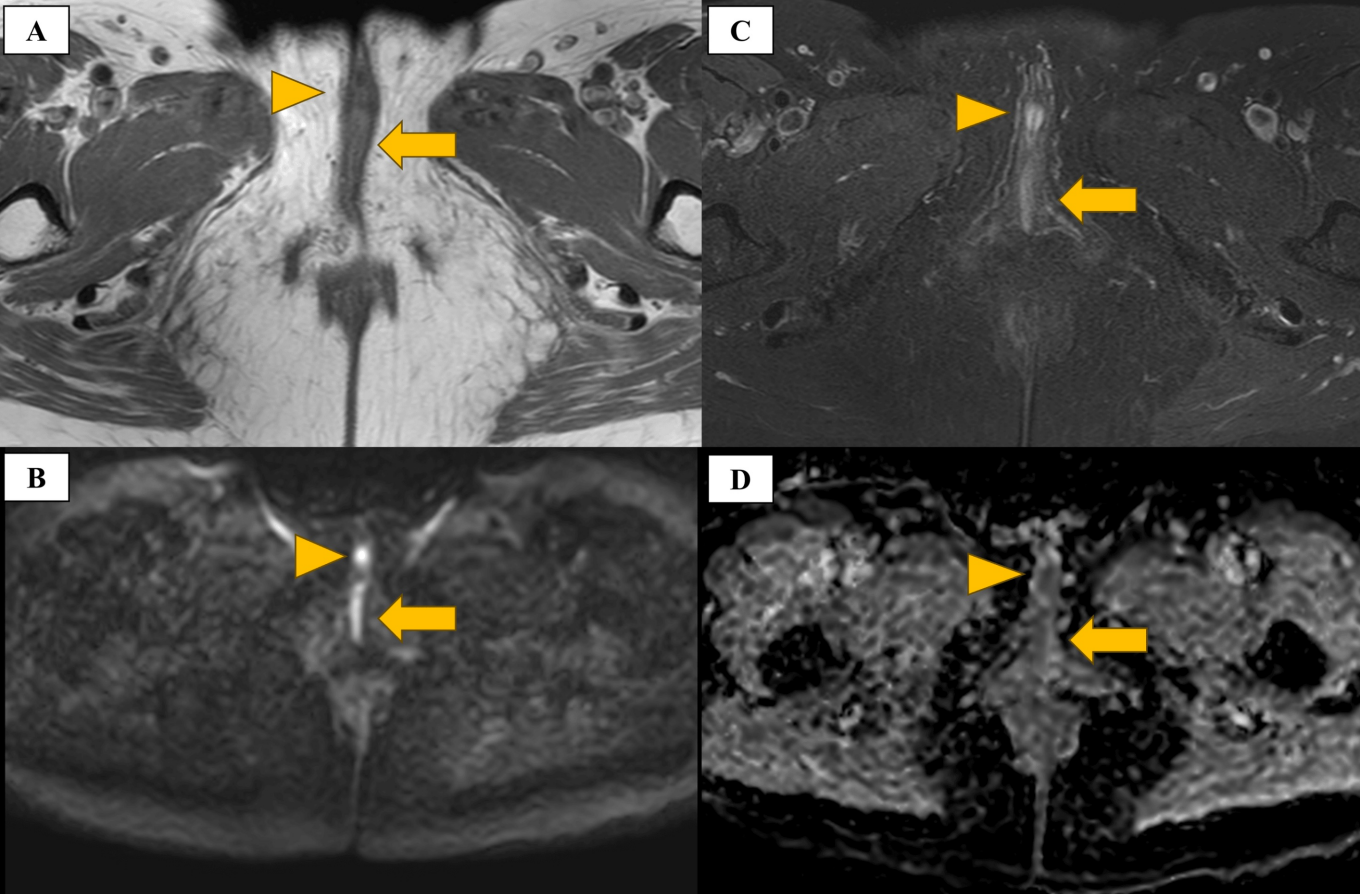
Pre-treatment MRI (A) T1-weighted image showing mild-high signal intensity in the vaginal wall (arrow) and urethra (arrowhead). (B) T2 fat-suppressed image showing high signal intensity in the vaginal wall (arrow) and urethra (arrowhead). (C) Diffusion-weighted image (b = 1000) showing high signal intensity in the vaginal wall (arrow) and urethra (arrowhead). (D) Apparent diffusion coefficient map showing a region of low signal intensity corresponding to a high signal intensity lesion on the diffusion-weighted image. MRI: magnetic resonance imaging

A 60-year-old woman visited the gynecology department of our institution with abnormal genital bleeding. Colposcopy revealed black pigmented lesions in the vulva, vaginal vestibule, fornix, and external urethral orifice (Figures 1A, 1B). Cystoscopy performed by a urologist revealed that the black pigmented lesions extended up to 2 cm from the external urethral orifice at the 4 to 8 o’clock position (Figure 1C). ^18^Fluorodeoxyglucose positron emission tomography/computed tomography (FDG-PET/CT) showed uptake in the vagina and urethra, but no lymph node or distant metastasis (Figure 2). Magnetic resonance imaging showed high signal intensity on T2 fat-suppressed images, a mild-high signal intensity on T1-weighted images, high signal intensity on diffusion-weighted images (b = 1000), and low signal intensity on the apparent diffusion coefficient map of the vaginal wall and urethra (Figure 3). A biopsy of the vaginal lesion was performed. Based on histopathologic findings of the vaginal lesion, a pathologist made a diagnosis of malignant melanoma. The patient was diagnosed with vaginal malignant melanoma and urethral invasion. A conference involving clinicians from the dermatology, gynecology, and urology departments was held, and considered the tumor to be unresectable. Because Ishiguro et al. reported favorable control with HDR-ICBT alone for vaginal melanoma [15], we decided to treat this case with HDR-ICBT alone.

**Figure 4:**
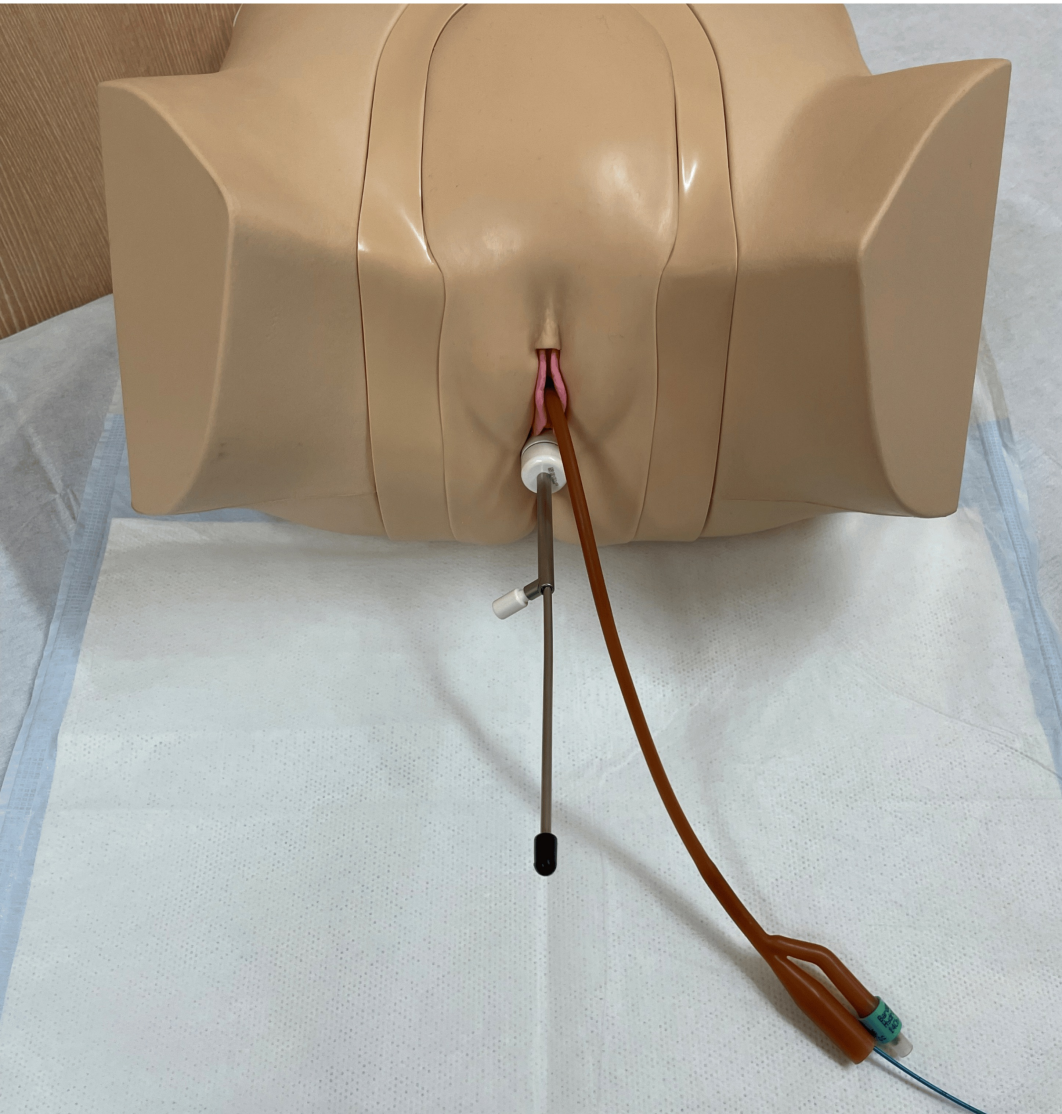
Model of the irradiation method A vaginal applicator is inserted into the vagina, a urethral catheter is inserted into the urethra, and a flexible catheter is placed inside the urethral catheter.

**Figure 5:**
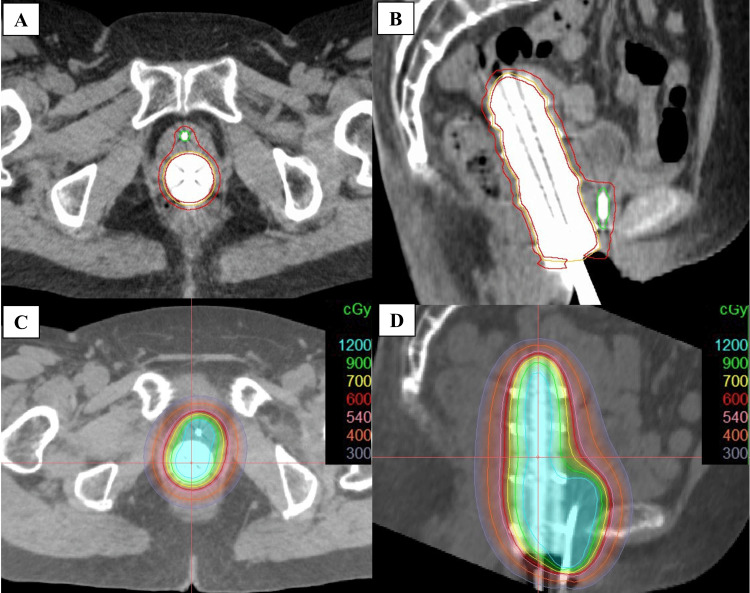
Axial and sagittal views of the CTV and dose distribution maps (A) Axial view of the CTV. (B) Sagittal view of the CTV. (C) Axial dose distribution map. (D) Sagittal dose distribution map CTV: clinical target volume

**Figure 6:**
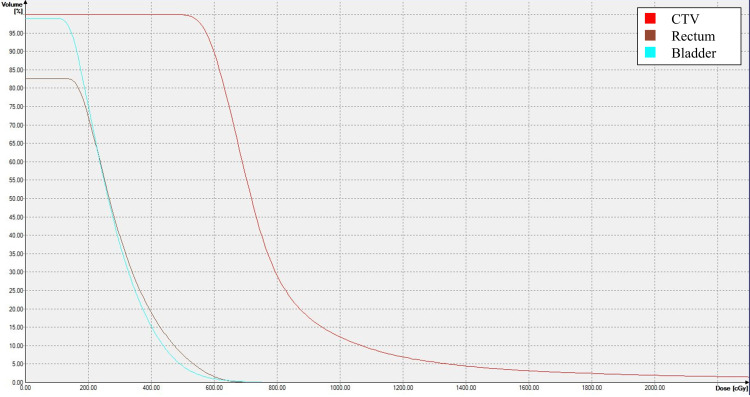
Dose-volume histogram The dose-volume histogram for the sixth treatment is shown. The dose applied to 90% of the CTV was 600 cGy. The minimum dose delivered to the most irradiated 2cc (D2cc) of the rectum was 597 cGy. The D2cc of the bladder was 525 cGy. CTV: clinical target volume

HDR-ICBT was performed in the lithotomy position. A vaginal applicator (Elekta, Stockholm, Sweden) was inserted into the vagina under direct visualization. A 16-French urethral catheter was inserted into the urethra. The balloon was inflated with 10 mL of water and fixed at the internal urethral orifice. A flexible catheter (Elekta) was inserted into the urethral catheter. This approach is presented using a model in Figure 4. The flexible catheter and urethral catheter were secured using medical tape (3M Center, Maplewood, MN, USA), and the insertion depth of the flexible catheter was marked and checked before and after CT imaging, prior to treatment planning, and after treatment delivery. The patient underwent CT imaging in the supine position using an Aquilion LB (Toshiba, Tokyo, Japan). We used the Eclipse treatment planning system (Varian Medical Systems, Palo Alto, CA, USA) to delineate the target volume and organs at risk because of its intuitive contouring tools and high accuracy. Because of our team’s extensive experience with Eclipse, we could accurately delineate the organs at risk and the clinical target volume (CTV). We calculated the dose using Oncentra Brachy (ver. 4.4; Elekta) and performed HDR-ICBT using a microSelectron HDR-V3 (Elekta). The CTV was defined as a depth of 5 mm from the vaginal wall and a depth of 5 mm from the urethral mucosa within 5 cm from the external urethral orifice. For each treatment fraction, CT was performed, and a new treatment plan was generated based on the updated anatomy. A total dose of 48 Gy (6 Gy × 8 fractions) was prescribed. The goal was to deliver 90% of the prescribed dose to the CTV. However, considering the rectal dose constraints, the median dose was 5.63 Gy (range 5.27-6.32 Gy). Table 1 shows the dose summary. The total treatment duration was 15 days. The schedule was determined based on the operator’s working days and the treatment facility’s availability. In practice, the eight fractions were delivered on Days 1, 2, 5, 6, 8, 12, 13, and 15. Representative dose-volume histograms and dose distributions are shown in Figures 5, 6, respectively. Starting 1 month after brachytherapy, the ICI pembrolizumab (200 mg/body) was administered once every 3 weeks.

**Table 1:** Parameters of the dose-volume histogram in one fraction CTV: clinical target volume D90 is defined as the dose applied to 90% of the volume of the CTV. D2cc is defined as the minimum dose delivered to the most irradiated 2 cc of an organ at risk; D0.5cc is defined as the minimum dose delivered to the most irradiated 0.5 cc of an organ at risk.

	CTV	Rectum	Bladder	Intestine	Urethra
Parameter	D_90_	D_2cc_	D_2cc_	D_2cc_	D_0.5cc_
Median (cGy, range)	563 (527–632)	546 (534–597)	468 (422–585)	388 (310–453)	2395 (1710–3526)

**Figure 7:**
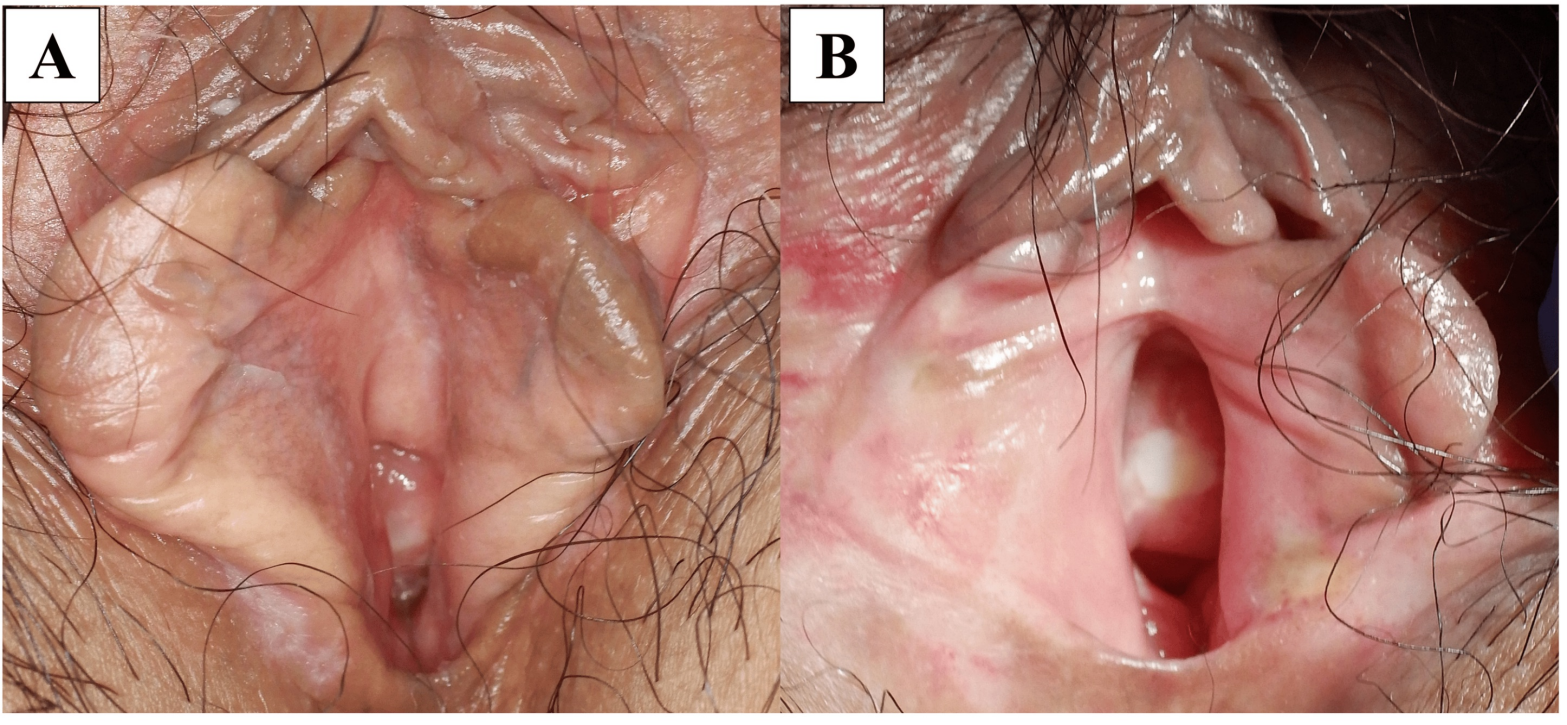
Follow-up imaging (A) Six months after treatment. All black pigmented lesions have disappeared. (B) Fifteen months after treatment. No recurrence is observed.

The black pigmented lesions on the vaginal wall showed signs of regression after the third HDR-ICBT session. The black pigmented lesion at the external urethral orifice began to regress after the fourth HDR-ICBT session and completely disappeared two weeks after the start of treatment. Cystoscopy performed three months after treatment revealed the disappearance of the urethral mucosal lesions. PET/CT performed three months after treatment showed no abnormal uptake in the urethra or vulva. At the six-month follow-up, all black pigmented lesions, including those on the vaginal wall, had completely disappeared (Figure 7A). No evidence of recurrent disease was noted on follow-up images at 15 months after treatment (Figure 7B). Adverse events were assessed using the Common Terminology Criteria for Adverse Events version 5. Grade 2 perivaginal dermatitis was observed at 6 weeks and Grade 3 vaginal stenosis was observed at 15 months; this was the only adverse event of Grade 3 or worse. No urethral stricture was observed.

## Discussion

This is the first report of transvaginal and transurethral HDR-ICBT for the treatment of malignant melanoma with urethral invasion. In this patient, we also used a urethral catheter, which is commonly used in clinical practice and facilitates the clinical application of the treatment approach. Urethral catheters have already been used in brachytherapy for prostate cancer. In particular, Watanabe et al. demonstrated that prostate cancer could be controlled by this approach without causing urethral stricture [16]. In our case, although treatment with a conventional applicator was not possible because the tumor invaded the urethra, we could effectively deliver the radiation dose to all tumor sites by using a urethral catheter. MMM is mainly treated with surgical resection. Radiotherapy, immunotherapy, chemotherapy, and ICIs are generally used as adjuvant therapies. Ishiguro et al. reported that HDR-ICBT for vaginal malignant melanoma achieved a 78% tumor reduction [15]. Based on their findings, we selected a dose regimen comprising 48 Gy in eight fractions. This treatment approach resulted in a complete response.

In this study, we selected pembrolizumab as part of the combination therapy. Backlund et al. reported that radiotherapy combined with an ICI improved OS from 7.2 to 18.2 months compared with radiotherapy alone in patients with metastatic malignant melanoma [10]. High radiation doses of 8-10 Gy induce cell death without exacerbating hypoxia or immunosuppression. This approach activates and promotes the proliferation of T cells against tumor antigens [17]. Although the prescribed dose per fraction was 6 Gy, >30% of the CTV received doses exceeding 8 Gy (Figure 6). Pembrolizumab administered after radiotherapy may have been effective due to radiation-induced expression of PD-L1.

Previous studies have reported effective tumor control with high-dose irradiation using stereotactic body radiotherapy or heavy-ion radiotherapy [18,19]. No severe adverse events have been reported with these modalities. However, compared with external beam radiotherapy, brachytherapy can apply a greater dose concentration to the tumor while minimizing exposure to organs at risk [13,14]. For primary vaginal malignant melanoma, brachytherapy achieved better local control than external beam radiotherapy alone. Therefore, brachytherapy should not be avoided, even in patients with adjacent organ invasion [20], and it may be an appropriate treatment option compared with other radiotherapy modalities.

This study has four limitations. First, it is a single-institution case report. Second, the follow-up period is short. Third, the effect of radiation alone could not be evaluated because brachytherapy was followed by ICI therapy. Prospective clinical trials are necessary to establish the efficacy of brachytherapy. Fourth, possible variations in the position of the flexible catheter within the urinary catheter, which could impact the dose distribution due to the steep dose gradient of HDR brachytherapy, were not quantitatively evaluated because our facility lacks an appropriate phantom.

## Conclusions

Transvaginal and transurethral intracavitary irradiation achieved a complete response in this patient with malignant melanoma and urethral invasion. The combination of transvaginal intracavitary irradiation using a vaginal applicator and transurethral irradiation using a urethral catheter and a flexible catheter allowed us to administer an adequate tumor dose to the target region.

## References

[1] Hida T, Idogawa M, Kato J (2024). Genetic characteristics of cutaneous, acral, and mucosal melanoma in Japan. Cancer Med.

[2] Tomizuka T, Namikawa K, Higashi T (2017). Characteristics of melanoma in Japan: a nationwide registry analysis 2011-2013. Melanoma Res.

[3] Zhang S, Zhang J, Guo J, Si L, Bai X (2022). Evolving treatment approaches to mucosal melanoma. Curr Oncol Rep.

[4] Yde SS, Sjoegren P, Heje M, Stolle LB (2018). Mucosal melanoma: a literature review. Curr Oncol Rep.

[5] Smith HG, Bagwan I, Board RE (2020). Ano-uro-genital mucosal melanoma UK national guidelines. Eur J Cancer.

[6] Shih MH, Chiang PJ, Meng E, Yu DS (2021). Primary melanoma of the vagina with urethral invasion: a case report and literature review. Taiwan J Obstet Gynecol.

[7] Todo Y, Okamoto K, Suzuki Y, Minobe S, Kato H (2016). Radicality of initial surgery for primary malignant melanoma of the vagina. Melanoma Res.

[8] Merten R, Strnad V, Karius A (2025). Definitive treatment for primary urethral cancer: a single institution's experience with organ-preserving brachytherapy. Brachytherapy.

[9] Cuccia F, D'Alessandro S, Blasi L, Chiantera V, Ferrera G (2023). The role of radiotherapy in the management of vaginal melanoma: a literature review with a focus on the potential synergistic role of immunotherapy. J Pers Med.

[10] Backlund E, Grozman V, Egyhazi Brage S, Lewensohn R, Lindberg K, Helgadottir H (2023). Radiotherapy with or without immunotherapy in metastatic melanoma: efficacy and tolerability. Acta Oncol.

[11] Patel SG, Prasad ML, Escrig M (2002). Primary mucosal malignant melanoma of the head and neck. Head Neck.

[12] Gutiérrez Miguélez C, Rodríguez Villalba S, Villafranca Iturre E (2023). Recommendations of the Spanish brachytherapy group of the Spanish Society of Radiation Oncology and the Spanish Society of Medical Physics for interstitial high-dose-rate brachytherapy for gynaecologic malignancies. Clin Transl Oncol.

[13] Schmid MP, Fokdal L, Westerveld H (2020). Recommendations from gynaecological (GYN) GEC-ESTRO working group - ACROP: target concept for image guided adaptive brachytherapy in primary vaginal cancer. Radiother Oncol.

[14] Gebhardt BJ, Vargo JA, Kim H (2018). Image-based multichannel vaginal cylinder brachytherapy for the definitive treatment of gynecologic malignancies in the vagina. Gynecol Oncol.

[15] Ishiguro A, Ogata D, Okuma K (2023). Malignant melanoma treatment using brachytherapy: two case reports and 15 case series. J Dermatol.

[16] Watanabe K, Kamitani N, Ikeda N (2024). Long-term outcomes of salvage transurethral high-dose-rate brachytherapy combined with external beam radiation therapy for anastomotic recurrence of prostate cancer after radical prostatectomy: a retrospective analysis. Brachytherapy.

[17] Demaria S, Golden EB, Formenti SC (2015). Role of local radiation therapy in cancer immunotherapy. JAMA Oncol.

[18] Stinauer MA, Kavanagh BD, Schefter TE (2011). Stereotactic body radiation therapy for melanoma and renal cell carcinoma: impact of single fraction equivalent dose on local control. Radiat Oncol.

[19] Murata H, Okonogi N, Wakatsuki M (2019). Long-term outcomes of carbon-ion radiotherapy for malignant gynecological melanoma. Cancers (Basel).

[20] Orton A, Boothe D, Williams N (2016). Brachytherapy improves survival in primary vaginal cancer. Gynecol Oncol.

